# Comparison of efficacy of different route of administration of chemotherapy on unresectable, advanced gastric cancer

**DOI:** 10.1186/1477-7819-10-162

**Published:** 2012-08-14

**Authors:** Caihua Zhang, Guoli Li, Chaogang Fan, Jian Xu, Jianmin Cao, Shen Liu, Ning Li

**Affiliations:** 1 Research Institute of General Surgery, Jinling Hospital, Clinical Medicine School of Nanjing University, 305 East Zhongshan Road, Nanjing, 210002, China; 2 Department of Medical Imaging, Jinling Hospital, Clinical Medicine School of Nanjing University, 305 East Zhongshan Road, Nanjing, 210002, China

**Keywords:** Gastric cancer, Neoadjuvant chemotherapy, Routes of drug administration

## Abstract

**Background:**

The aim of this study was to compare the efficacy of two neoadjuvant chemotherapies (FLEEOX and XELOX) with different routes of administration for unresectable gastric cancer.

**Methods:**

A total of 85 patients with unresectable gastric cancer hospitalized from January 2007 to December 2009 received neoadjuvant chemotherapy. The FLEEOX group (48 patients) received the FLEEOX regimen(fluorouracil, leucovorin, http://epirubicin, epotoside, and oxaliplatin), which combined arterial with venous administration for one or two cycles, while the XELOX group (37 patients) received XELOX (capecitabine plus oxaliplatin) via venous administration for two to four cycles. The clinical response and overall survival of the two groups were compared.

**Results:**

In the FLEEOX group, the clinical response rate (RR) of chemotherapy was 85.4% (41 of 48 patients) and the median survival time was 25 months. The 1-year and 2-year disease-free survival (DFS) rates were 85.4% and 45.8%, respectively. In the XELOX group, the clinical RR was 59.5% and the median survival time was 9 months, while the 1-year and 2-year survival rates were 35.2% and 8.3%, respectively. The clinical RR, the R0 resection rate, the median survival time, and the 1-year and 2-year DFS rates were significantly better (*P <* 0.05) in the FLEEOX group than in the XELOX group. In addition, there were no significant differences in the rates of toxic and adverse reactions or post-operative complications between the two groups.

**Conclusions:**

For patients with a preoperative diagnosis of unresectable gastric cancer, the efficacy of the FLEEOX regimen, which combines arterial with venous administration, was better than that of the XELOX regimen, using venous administration only. This combination of arterial and venous administration could be useful for improving the efficacy of neoadjuvant chemotherapy for gastric cancer.

## Background

Gastric cancer is the second most common cancer in the world. Nearly 41% of the global gastric cancer cases occur in China [1], and the vast majority of cases in China present as advanced gastric cancer. The efficacy of surgical treatment for advanced gastric cancer is not high [2], and efforts are being made to improve treatments for gastric cancer. In recent years, a number of clinical studies (including the Medical Research Council Adjuvant Gastric Infusional Chemotherapy (MAGIC) trial) have shown that preoperative chemotherapy can improve the outcomes in advanced gastric cancer [3], which opens up a new avenue for treating this cancer. At present, the approaches and methods of post-operative adjuvant chemotherapy of gastric cancer are generally used for preoperative chemotherapy as well.

Several chemotherapy regimens are available. The XELOX regimen has the advantages of easy administration and two effective drugs that have been used in neoadjuvant chemotherapy for advanced gastric cancer [4,5], namely, oxaliplatin and capecitabine [6-8]. Based on the FLEP (5-FU, leucovorin, etoposide, and cisplatin) regimen. After the exploring of the preoperative chemotherapy which combines arterial with venous administration since December 2002 [9,10] we obtained a very satisfactory efficacy rate with the FLEEOX regimen for unresectable, advanced gastric cancer. The FLEEOX regimen is a combination of venous and regional chemotherapy, comprising continuous intravenous infusion 5-fluorouracil and leucovorin, followed by intra-aterial infusion of epirubicin, etoposide, and oxaliplatin. In previous studies, the initial radiological response rate (RR) with this regimen was about 80% [11].

In this study, we compared venous administration of XELOX versus combined arterial and venous administration of FLEEOX, and assessed the short-term efficacy of both groups.

## Methods

### Ethics approval

The study was approved by Ethics committee of Jinling Hospital and informed consent was obtained from all patients.

The study enrolled patients with unresectable, advanced gastric cancer who were hospitalized from January 2007 to December 2009. Cancers meeting the following criteria were diagnosed as unresectable gastric cancer:1) strongly suspicious for stage III or IV metastasis of lymph nodes by enhanced CT examination; 2) tumor infiltration and encompassment of major blood vessels (for example, hepatic artery, celiac artery, and portal vein); 3) distant metastasis (for example, liver metastasis). The inclusion criteria were:age35 to75 years; Eastern Cooperative Oncology Group (ECOG) score of 0 to 2; no previous history of curative or palliative surgery, radiotherapy or chemotherapy; no serious cardiovascular, liver, or kidney disease; and acceptance of chemotherapy and interventional chemotherapy. The exclusion criteria were: pregnancy or breast-feeding; presence of peritoneal seeding or distant metastasis except in the liver; presence of other malignant tumors; history of curative or palliative surgery, radio therapy, or chemotherapy; history of upper gastrointestinal bleeding, perforation, obstructive jaundice, severe infections, or other serious complications; history of any serious or uncontrollable venous diseases; or sensitivity to any of the chemotherapy drugs.

In total, 85 patients (62 men, 23 women, 61 ± 13 years, range 35 to 75 years) were enrolled in the study. The diagnosis of gastric cancer was confirmed by histopathological examination of an endoscopic biopsy sample. The clinical staging of all cases were confirmed by CT and endoscopic ultrasonography before treatment. The relationship between the tumor and major blood vessels or organs in its proximity, and the extent of lymph-node metastasis were assessed by multi-slice spiral CT and endoscopic ultrasonography.

### Preoperative chemotherapy

The selected cases were randomly divided into two groups to receive the appropriate preoperative chemotherapy regimens: 48 patients received the FLEEOX regimen, which combined arterial with venous administration, and 37 patients received the XELOX regimen, which was given by venous administration only.

The FLEEOX regimen consisted of a slow intravenous infusion of 5-fluorouracil(5-FU) 370 mg/m^2^over 5 days and an intravenous infusion of calcium folinate 200 mg/m^2^over 5 days. Following this, oxaliplatin 120 mg/m^2^, epirubicin 30 mg/m^2^, and etoposide 70 mg/m^2^ were injected into the tumor site on days 6 and 20. Patients received one or two cycles of this regimen; for those patients receiving two cycles, the second cycle was administered after an interval of 5 weeks. The arterial administration was performed using the Seldinger method. After intubation of the celiac artery, a catheter was inserted into the blood supply of the tumor depending on the tumor site; for example, the catheter was inserted into the left gastric artery for cancer of the upper and central stomach, and through the hepatic and gastroduodenal arteries into the right gastroepiploic artery for cancer of the lower part of the stomach. First, half the drug volume was injected into the artery supplying the tumor, then the remaining drug was injected into the celiac artery, except for patients with accompanying liver metastases, for whom the second half of the drug was injected into the metastatic focus in the liver. Lipiodol embolization was also performed for the latter patients.

The XELOX regimen comprised intravenous infusion of oxaliplatin 130 mg/m^2^over 2 hours on day 1, followed by capecitabine 1000 mg/ m^2^ orally twice daily for 2 weeks. This cycle was repeated once every 3 weeks, and patients were given two to four cycles.

### Evaluation criteria for efficacy and adverse events

The efficacy was evaluated by CT after three cycles of intervention treatment in the FLEEOX (combined arterial and venous administration), and three cycles in the XELOX (intravenous administration) group. Patients whose tumors were deemed resectable would have their chemotherapy stopped and surgery performed. Patients whose tumors were considered unresectable would continue chemotherapy for two cycles (FLEEOX group) or four cycles (XELOX group), after which the efficacy was again evaluated by CT. At this point, any patients with resectable tumors would undergo surgery. The study would be stopped for any patients whose tumors were not resectable.

The efficacy was evaluated by two radiologists with using the Response Evaluation Criteria In Solid Tumors (RECIST) criteria [12]. Tumors were evaluated as follows: complete disappearance of the tumor was considered to be complete response (CR); a decrease of more than 30% in tumor size to be partial response (PR), and an increase of more than 20% in tumor size as progressive disease (PD), neither sufficient shrinkage to qualify for partial response nor sufficient increase to qualify for progressive disease was considered stable disease (SD). Clinical RR was calculated as: (CR + PR)÷measurable number of cases) × 100%. Surgery (mainly D2 gastrectomy) was carried out within 2 weeks of the end of preoperative chemotherapy for patients with resectable gastric cancer. D3 gastrectomy was performed for patients with tumors andN3 lymph-node metastasis.

### Adverse events

Patients were closely monitored for liver and kidney function, bone-marrow hematopoiesis, gastrointestinal reactions, and related adverse events during the treatment. Toxic reactions were evaluated using the National Cancer Institute Common Toxicity Criteria (version 3.0) and compared between two groups.

### Follow-up

After chemotherapy, patients attended a clinical follow-up review every 3 months, which included physical examination, routine blood investigations, assessment of liver and kidney function, tumor markers, abdominal CT, and chest X-ray. Gastroscopy was performed once every year.

### Statistical analysis

The primary objective indicators were clinical objective RR (CR + PR), 1-year and 2-year disease-free survival (DFS) rates, and median survival time. The survival rate was calculated by the Kaplan-Meier method. Secondary indicators were R0 resection rate, characteristics of the resected specimen, and toxic reactions. Overall survival (OS) time was recorded from first chemotherapy to death or the last follow-up visit. Comparison between the two groups was performed using the χ² test. Data were analyzed using the SPSS statistical software (version 17.0; SPSS Inc., Chicago, IL, USA). P < 0.05 was considered significant.

## Results

The age and sex distribution and physical condition of the patients were similar in both groups (median age of the patients was 62 years in the FLEEOX group and 59 years in the XELOX group; Table 1). Both groups were also comparable with regard to tumor site, degree of tumor differentiation, and stage of tumors (*P >* 0.05) (Table 1).

**Table 1:** Pre-treatment characteristics of patients enrolled (n = 85)

	**FLEEOX , n = 48**	**XELOX, n = 37**	***P*value**
Patients, n	48	37	
Age, years)	38 to 73	38 to 75	
Gender, n (%)			
Male	35 (72.9)	27 (73.0)	1.00
Female	13 (27.1)	10 (27.0)	
ECOG^1^			
0	29 (60.4)	21 (56.8)	0.94
1	13 (27.1)	11 (29.7)	
2	6 (12.5)	5 (13.5)	
Site of lesion			
Cardia	19 (39.6)	17 (45.9)	0.82
Gastric body	15 (31.3)	11 (29.7)	
Gastric antrum	14 (21.1)	9 (24.4)	
Degree of differentiation			
Severe	3 (6.3)	2 (5.4)	0.57
Moderate	13 (27.1)	14 (37.8)	
Mild	32 (66.6)	21 (56.8)	
Pre-treatment stage, n (%)			
III	20 (41.7)	21 (56.8)	0.19^1^
IV	28 (58.3)	16 (43.2)	
Reasons for non-resection			
Tumor and metastatic lymph nodes with encompassment of major blood vessels	44 (91.7)	35 (94.6)	0.74
Liver metastasis	1 (2.1)	1 (2.7)	
NO.16 group of lymph nodes	3 (6.3)	1 (2.7)	

Abbreviations: *ECOG*, Eastern Cooperative Oncology Group; *FLEEOX*, fluorouracil, leucovorin, http://epirubicin, epotoside and oxaliplatin; *XELOX*. capecitabine plus oxaliplatin.

^1^Significant.

### Response rate of chemotherapy

Of the forty-eight patients in the FLEEOX group, six patients were rated as having CR, thirty-five as PR, five as SD, and two as PD; the clinical RR was 85.4%. For the thirty-seven patients in the XELOX group, the figures were one, twenty, twelve and four, respectively, and the clinical RR was 59.5%. There was a significant difference in RR (*P <* 0.02) between the two groups (Table 2).

**Table 2:** Response to treatment of chemotherapy of the patients in both groups

Response rates	**FLEEOX, n = 48**	**XELOX,n = 37**
CR, n (%)	6 (12.5)	1 (2.7)
PR, n (%)	35 (72.9)	20 (54.1)
SD, n (%)	5 (10.4)	12 (32.4)
PD, n (%)	2 (4.2)	4 (10.8)

Abbreviations: *CR*, complete response; *FLEEOX*, fluorouracil, leucovorin, http://epirubicin, epotoside and oxaliplatin; *PD*, progressive disease; *PR*, partial response; *SD*, stable disease; *XELOX*. capecitabine plus oxaliplatin.

### Surgery

In the FLEEOX group, the tumors were evaluated as resectable in nine patients after the second treatment cycle and in thirty-two patients after the third cycle. Of the remaining seven patients, six were evaluated as invalid or having disease progression, and one patient refused surgery. Of the forty-one patients approved for surgery, thirty-eight underwent D2 gastrectomy and three underwent D3 gastrectomy. The gastrectomy was a distal subtotal gastrectomy in twenty patients and a total gastrectomy in twenty-one patients. Thirty-six of the patients underwent R0 resection (thus theR0 resection rate was 75%; 36/48), while four patients underwent R1 resection and one underwent R2 resection.

The thirty-seven patients in the XELOX group received two (n = 5), three (n = 11) or four (n = 21) cycles of chemotherapy. Sixteen patients were evaluated as invalid or having disease progression, and thus could not undergo surgery. The remaining twenty-one patients who were approved for surgery all underwent D2 gastrectomy, with twelve operations being distal subtotal gastrectomy and nine being total gastrectomy. Seventeen patients underwent R0 resection (R0 resection rate of 45.9% (17/37)), while one patient underwent R1 resection and three underwent R2 resection.

There was a significant difference between the two groups in the R0 resection rate (P < 0.001)(Table 3).

**Table 3:** Rates of surgery in both groups

	**FLEEOX, n = 48**	**XELOX, n = 37**
R0, n (%)	36 (75.0)	17 (45.9)
R1, n (%)	4 (8.3)	1 (2.7)
R2, n (%)	1 (2.1)	3 (8.1)
Inoperable, n (%)	7 (14.5)	16 (43.3)
Refused surgery, n (%)	1 (2.1)	0 (0)

*FLEEOX*, fluorouracil, leucovorin, http://epirubicin, epotoside and oxaliplatin; *XELOX*. capecitabine plus oxaliplatin.

### Changes in surgical specimens

**Figure 1:**
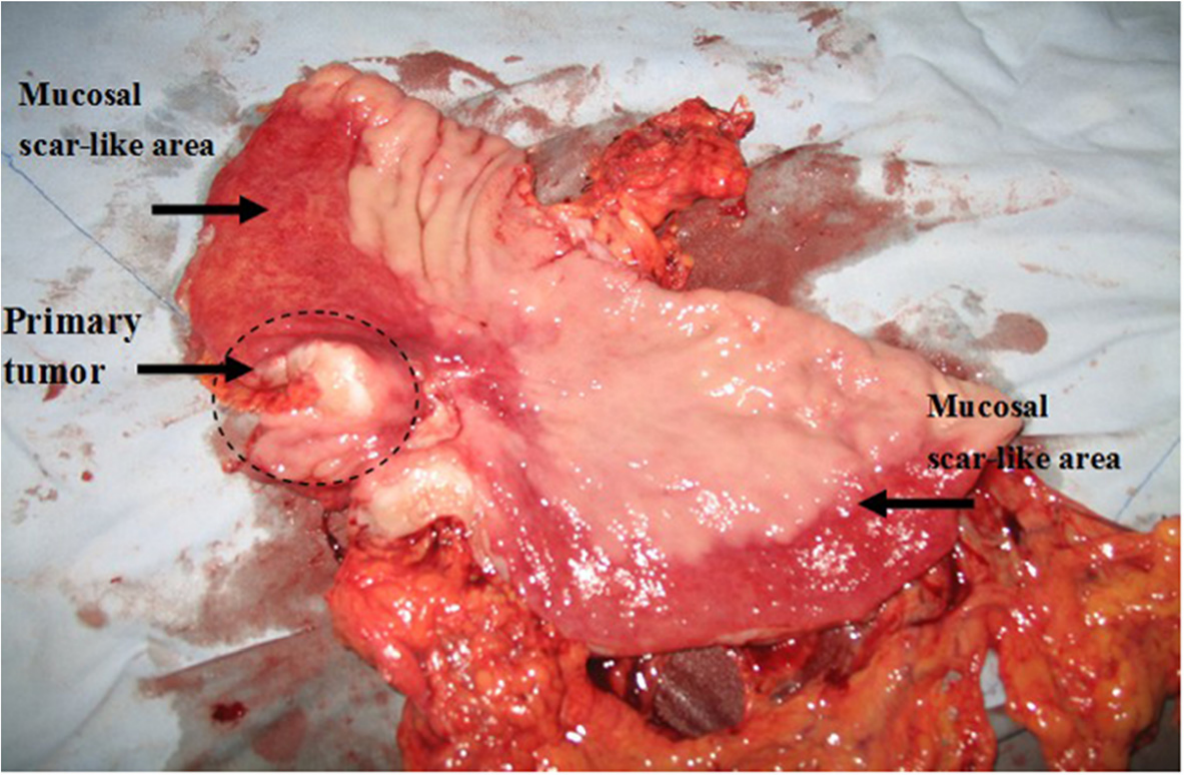
**Post-operative specimen.** The mucous membrane shows scar-like changes after chemotherapy in areassupplied by the right gastroepiploic artery, but the gastric mucosa outside the chemotherapy delivery area was normal.

Upon intra-operative and post-operative observation of the primary tumors and metastatic lymph nodes, atrophic changes were found in 27 primary tumors in FLEEOX group, which occurred as caseous necrosis and scar-like changes in the corresponding lymph nodes. Necrosis of the gastric mucosa was visible in the peri-tumoral arterial administration area in 17 patients. Severe cases showed obvious scar-like changes (Figure 1).

Atrophic changes were found in 13 primary tumors in the XELOX group, which produced caseous necrosis on the corresponding lymph nodes, but the gastric mucosa in the peri-tumoral area was normal in all patients.

### Post-operative complications

Between the two groups, there were 76 patients who received D2 gastrectomy. There were no deaths due to the surgery. There were seven (16.3%) instances of post-operative complications in the FLEEOX group (two cases of intestinal obstruction and two cases of abdominal infection, and one case each of anastomotic leakage, pneumonia, and wound infection), and six (18.1%) in the XELOX group (two cases of abdominal infection, and one case each of intestinal obstruction, anastomotic leakage, pneumonia, and wound infection). There was no significant difference in the incidence rate of postoperative complication of the patients in two groups (P >0.05), and all patients recovered with conservative treatment.

### Toxicity

In total, there were 207 completed cycles of chemotherapy for the two groups. No treatment termination or death occurred as a result of toxic reactions. During the chemotherapy, there were different degrees of toxic and adverse reactions in the two groups. The reactions were mainly myelosuppression, liver dysfunction, and gastrointestinal reactions. With regard to bone-marrow suppression, the levels of hemoglobin, white blood cells, and platelets decreased in some patients within both group. The most common symptom was leukopenia (n = 30; 62.5%) in the FLEEOX group and anemia (n = 24; 64.9%) in the XELOX group. Alanine aminotransferase (ALT) and aspartate aminotransferase (AST), which are markers of liver injury, increased in nine (18.8%) and eleven patients (22.9%), respectively, in the FLEEOX group, and in nine (24.3%) and eight patients (21.6%) in the XELOX group. The commonest digestive-tract reactions in both groups were varying degrees of nausea and vomiting, with 38 patients (79.2%) in the FLEEOX group reporting nausea,22(57.9%) patients vomiting, and 19 patients (51.4%) in the XELOX group reporting vomiting,25(67.7%) patients nausea. Toxic neurological reaction occurred in 21 patients (56.8%) in the XELOX, but in only 11 patients (22.9%) in the FLEEOX group. For most patients, there were only minor changes in renal function, with no marked change in levels of creatinine or urea nitrogen before and after chemotherapy in either groups. There was no significant difference in the number of toxic reactions between the two groups (P >0.05) (Table 4).

**Table 4:** Comparison of toxic reactions of chemotherapy in both groups

**Toxic reactions**	**FLEEOX**	**XELOX**
Hematologic		
Anemia,n(%)		
Grade 0, 1 or 2	45 (94.8)	37(100)
Grade 3 or 4	3 (5.2)	0 (0)
Leukopenia,n(%)		
Grade 0, 1 or 2	46 (95.8)	36 (97.3)
Grade 3 or 4	2 (4.2)	1(2.7)
Neutropenia,n(%)		
Grade 0, 1 or 2	48 (100)	37 (100)
Grade 3 or 4	0 (0)	0(0)
Thrombocytopenia,n(%)		
Grade 0, 1 or 2	45 (94.8)	35 (94.6)
Grade 3 or 4	3 (5.3)	2 (5.4)
Abnormal AST,n(%)		
Grade 0, 1 or 2	44 (91.7)	37 (100)
Grade 3 or 4	4 (8.3)	0 (0)
Abnormal ALT,n(%)		
Grade 0, 1 or 2	44 (91.7)	37 (100)
Grade 3 or 4	4 (8.3)	0 (0)
Nonhematologic		
Nausea,n(%)		
Grade 0, 1 or 2	43 (89.6)	36 (97.3)
Grade 3 or 4	5 (10.4)	1 (2.7)
Vomiting,n(%)		
Grade 0, 1 or 2	45 (94.8)	35 (94.6)
Grade 3 or 4	3 (5.2)	2 (5.4)
Neurotoxicity,n(%)		
Grade 0, 1 or 2	48 (89.6)	36 (97.3)
Grade 3 or 4	0	1 (2.7)
HFSR ,n(%)		
Grade 0, 1 or 2	45 (94.8)	37 (100)
Grade 3 or 4	3 (5.2)	0 (0)
Renaldysfunction,n(%)		
Grade 0, 1 or 2	48 (89.6)	37 (100)
Grade 3 or 4	0	0 (0)

### Survival and disease-free survival

**Figure 2:**
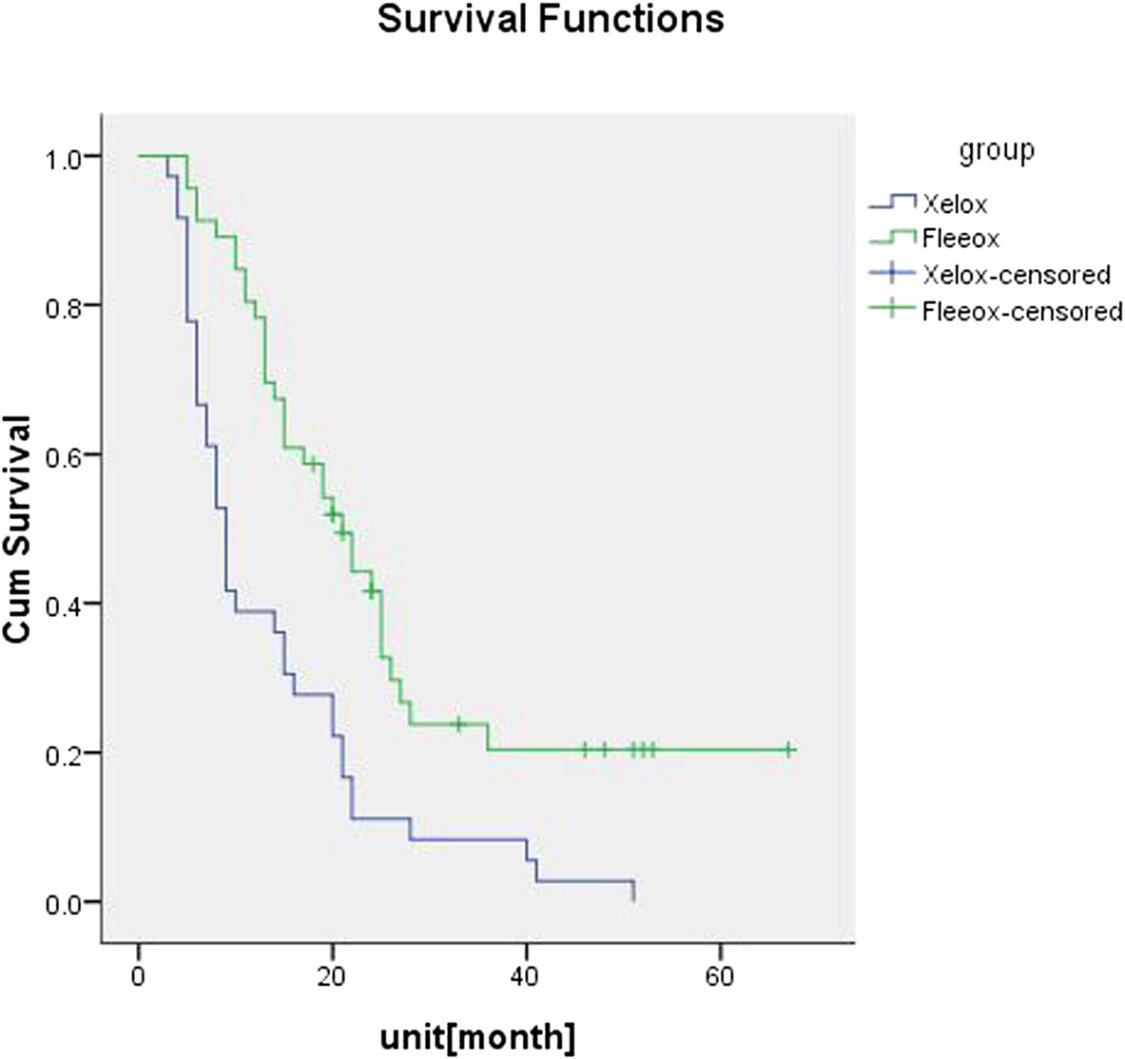
**Survivalin both groups.** The median survival time was 25 months in the FLEEOX group and 9 months in the XELOX group.

**Figure 3:**
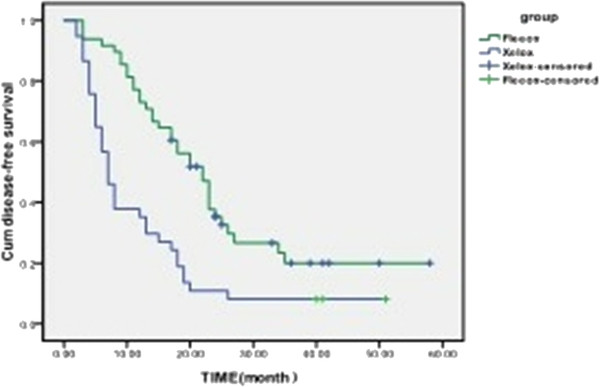
Disease-free survival curves for both groups.

The survival time of the 85 patients was calculated. The median survival time was 25 and 9 months in the FLEEOX group and the XELOX group, respectively, which was significantly different (*P <* 0.001) (Figure 2). The 1-year and 2-year DFS rates were 85.4% and 45.8%, respectively, in the FLEEOX group, and 35.2% and 8.3%, respectively, in the XELOX group. The 1-year and 2-year DFS rates were 83.3% (40/48) and 33.3% (16/48) respectively in the FLEEOX group, with the number of recurrences in R0 patients being two and eighteen in the first year and second years, respectively. For the XELOX group, the 1-year and 2-year DFS rates were 33.3% (16/48) and 5.4%(2/37)respectively, and the number of recurrences in R0 patients were three and twelve in the first and second years, respectively. There was a significant difference in DFS rates between the two groups (P < 0.05) (Figure 3).

## Discussion

The result of surgical treatment of advanced gastric cancer is often less than ideal [13]. The prognosis of unresectable gastric cancer with severe lymph-node metastasis or distant metastasis is poor, with a survival time of only 6 to 12 months [14-17]. Many clinical studies have shown that chemotherapy can downstage the tumor, eliminate micrometastases, and make some unresectable gastric cancers resectable, thereby prolonging the survival time of patients [18-21]. Therefore, preoperative chemotherapy of gastric cancer has become an important part of cancer treatment, and identifying effective chemotherapy regimens has become the focus of clinical research into gastric cancer.

Based on the FP regimen, 5-FU and cisplatin are instead of capecitabine and oxaliplatin which have the similar efficacy but lower toxicity of chemotherapy [22,23] for XELOX regimen, making XELOX regimen easier to accept in clinical practice, which is the more commonly used method of venous administration at present. Park *et al*. showed that the clinical RR to the XELOX regimen to the patients with advanced gastric cancer was 63%, and median survival time was 11.9 months [24]. In this study, we obtained similar results, with a clinical RR of 59.5%, and a median survival time of 9 months.

The development of the EAP regimen (etoposide, doxorubicin, and cisplatin) greatly improved the efficacy of preoperative chemotherapy for gastric cancer greatly, but this regimen is highly toxic. Some authors reported the use of arterial infusion for this regiment, but it did not significantly reduce toxicity. Nakajima *et al*., used arterial infusion of etoposide and cisplatin, in addition to intravenous infusion of 5-FU and leucovorin. The FLEP regimen, which combines arterial with venous administration, is a development of the EAP (etoposide, doxorubicin and cisplatin) regimen, with excellent efficacy [18]. Drawn from the experience of Nakajima *et al*., the FLEEOX regimen uses oxaliplatin , which is less toxic than cisplatin, and thus reduce the toxicity of arterial administration. The adding of epirubicin helps to form the EEOX regiment, which uses arterial administration, and is similar to the EAP regimen. 5-FU is a time-dependent drug, while epirubicin and oxaliplatin are concentration-dependent drugs, thus a slow intravenous infusion of 5-FU can maintain its effective time, while local arteiral infusion of EEOX can maintain the concentrations. The pharmacological effect of two different types of drug can be enhanced by the different routes of administration, which can take the clinical RR up to 85.4%. In the current study, the R0 resection rate and survival rate of the FLEEOX group were substantially compared with the XELOX group, but the toxicity reactions did not increase significantly.

We also found that a considerable number of the resected specimens in the FLEEOX group, showed evidence of necrosis and scarring in the gastric mucosa within the region of administration, probably due to high concentrations of drug. This shows that the FLEEOX regimen using local arterial administration has a very intense effect on local tissues. This type of damage did not occur in the XELOX group, probably because it used venous administration only and the drug concentrations were lower than FLEEOX.

The rationale behind drug combinations is to use several drugs acting on different parts of the cell cycle to improve the efficacy, produce a synergistic beneficial effect, and avoid deleterious combinations of the toxic effects of the drugs. After years of research, the currently available chemotherapy drugs have been played the synergistic effect adequately The current regimens have reached the limit of the synergistic effect [8,25-28], and thus, without the development of more effective drugs, it is difficult to greatly improve the efficacy of the available chemotherapeutic regimens for gastric cancer. Thus, in the current study, we investigate a preoperative chemotherapy regimen that combined arterial and venous administration, that is, using an intravenous slow infusion of a time-dependent drug to optimize its effective time, along with local arterial administration of a concentration-dependent drug to maintain its local concentration. The different routes of administration can enhance the pharmacological effect of the drugs by exploiting their different actions. By using an optimal combination of administration routes, it should be possible not only to enhance the pharmacological effect but also to use combinations of five drugs, which could greatly improve the efficacy of the regimens.

## Conclusions

Our results show that for patients with a preoperative diagnosis of unresectable gastric cancer, the efficacy of the FLEEOX regimen, which combines arterial with venous administration, was better than that of the XELOX regimen, using venous administration only. The FLEEOX regimen is a safe and promising regimen for preoperatively treatment of advanced gastric cancer.

## Competing interests

The authors declared that they have no competing interests.

## Authors’ contribution

GLL was the lead author and surgeon for all of the patients. SL undertook the literature research. CHZ and SL gathered information on the patients and contributed to writing of the paper.CGF and NL were the co-surgeon on the cases. JX, JMC and SL performed the data and statistical analysis. CHZ prepared the manuscript. All authors read and approved the final manuscript.
